# PARP-1 Controls Immunosuppressive Function of Regulatory T Cells by Destabilizing Foxp3

**DOI:** 10.1371/journal.pone.0071590

**Published:** 2013-08-19

**Authors:** Pin Zhang, Takashi Maruyama, Joanne E. Konkel, Brittany Abbatiello, Brian Zamarron, Zhao-qi Wang, WanJun Chen

**Affiliations:** 1 Mucosal Immunology Unit, OIIB, National Institute of Dental and Craniofacial Research, NIH, Bethesda, Maryland, United States of America; 2 Leibniz Institute for Age Research – Fritz Lipmann Institute e.V. 07745, Jena, Germany; University Heart Center Freiburg, Germany

## Abstract

Poly (ADP-ribose) polymerase-1 (PARP-1) is a nuclear enzyme and transcription factor that is involved in inflammatory response, but its role in T cell response remains largely unknown. We show here that PARP-1 regulates the suppressive function of CD4^+^CD25^+^Foxp3^+^ regulatory T cells (Tregs). Specifically, Tregs in mice with a null mutation of the PARP-1 gene (PARP-1^–/–^) showed significantly stronger suppressive activity than did wild-type Tregs in culture. We elucidate that this enhanced suppressive function is attributed to sustained higher expression of Foxp3 and CD25 in PARP-1^−/−^ Tregs. Furthermore, in PARP-1^−/−^ Tregs, Foxp3 protein shows substantially higher levels of binding to the conserved non-coding DNA sequence 2 (CNS2) at the *foxp3* gene, a region important in maintaining Foxp3 gene expression in Tregs. Thus, our data reveal a role for PARP-1 in controlling the function of Tregs through modulation of the stable expression of Foxp3.

## Introduction

CD4^+^CD25^+^Foxp3^+^ regulatory T cells (Tregs) are essential in the induction and maintenance of immune tolerance and therefore play a critical role in the prevention and inhibition of inflammation and autoimmunity [1]–[8]. Tregs regulate immune responses by multiple yet non-exclusive mechanisms including (but not limited to) cell-cell contact involving CTLA-4 and immunoregulatory cytokines such as transforming growth factor-beta (TGF-β1) and IL-10 [8]–[13]. In addition, the surface expression of CD25 may also participate in Tregs-mediated immunoregulation as high constitutive levels of CD25 on Tregs allow them to consume IL-2 produced by responding T cells and thereby inhibiting T cell proliferation and differentiation [14], [15]. Expression of Foxp3 has been shown to be sufficient to confer the regulatory phenotype and deletion or reduction of Foxp3 in CD4^+^CD25^+^ Tregs diminish their suppressive ability [16], 17. Despite this, the underlying molecular mechanisms that bestow this sufficient and stable expression of Foxp3 remain elusive. Recent epigenetic studies have suggested that the non-coding DNA elements region 2 (CNS-2) plays an important role in maintaining the expression of Foxp3 in Tregs [18], but the factors influencing Foxp3 binding to this CNS2 region remain largely unknown.

Poly(ADP-ribose) polymerase-1 (PARP-1) is a nuclear enzyme that is conventionally linked to DNA repair, and can be activated by DNA strand breaks and kinks [19]–[21]. Recently, however PARP-1 has also been shown to function as a transcription factor involved in a number of gene transcription networks including NF-κB and the autoimmune regulator (AIRE) gene [22]. Inhibition of PARP-1 activity by its inhibitors or by gene mutation in mice has been shown to lead to suppression of chronic inflammation and autoimmunity [23]–[26]. Of note, PARP-1 deletion leads to suppression of innate immunity by inhibiting NF-κB activation including decrease in TNF and inducible NO synthesis [25], [27], The role of PARP-1 in T cell immune responses remains elusive, as CD4^+^CD25^+^Foxp3^+^ Tregs are instrumental in regulation of immune responses and suppression of autoimmunity, we hypothesized that PARP-1 played a role in the suppressive function of Tregs.

Indeed, here we show that PARP-1 controls the suppressive activity of CD4^+^CD25^+^Foxp3^+^ Tregs by regulating the expression levels of Foxp3. Tregs from PARP-1^−/−^ mice exhibited a much more robust immunosuppressive function to TCR-mediated T cell proliferative response compared to WT control Tregs in cultures. This increased suppressive function was largely due to the higher and more stable expressions of Foxp3 and surface CD25 in PARP-1^−/−^ Tregs. Importantly, we identified that substantially more Foxp3 is recruited to the CNS2 region of *Foxp3* gene in PARP-1^−/−^ Tregs than in WT Tregs. Collectively these data reveal a role for PARP-1 as a negative regulator of Foxp3^+^ Tregs suppressive capacity.

## Materials and Methods

### Mice

We obtained the mice from Dr. Wang's lab in Germany as a gift. The generation of PARP-1 knockout mice (PARP-1^−/−^) has been described. Genotypes were determined by PCR. 6–8 weeks PARP-1^−/−^ mice on 129/Sv background and age-matched wild type (WT) control mice were used in the experiments and were bred and maintained under specific, pathogen-free conditions in the animal facilities of the National Institutes of Health (NIH). All animal studies were performed according to NIH guidelines for use and care of live animals and approved by Animal Care and Use Committee of National Institute of Dental and Craniofacial Research (NIDCR).

### Antibodies and Reagents

Monoclonal antibodies anti-CD3 (clone 145-2C11), anti-CD28 (clone 37.51), anti-CD16/CD32 (clone 93), allophycocyanin (APC)-conjugated anti-CD25 (clone PC61.5), Fluorescein isothiocyanate (FITC)-conjugated anti-CD4 (clone GK1.5), Peridinin chlorophyll protein (Percp)-conjugated anti-CD8 (clone 53–6.7) were purchased from BD Biosciences. Mouse CD4^+^CD25^+^ T cell isolation Kit was obtained from Miltenyi Biotec (Auburn, CA). APC-conjugated anti-Foxp3 (clone FJK-16s) and Rat IgG2a Isotype control were purchased from eBioscience (San Diego, CA). Carboxyfluorescein succinimidyl ester (CFSE) was purchased from Invitrogen (Carlsbad, CA). TGF-β receptor I kinase inhibitor II was purchased from Calbiochem (Darmstadt, Germany) and Anti-IL10 receptor antibody was obtained from R&D Systems. IL-2, IL-4, IFN-γ and TNF ELISA kits were purchased from Biolegend, IL-6, IL-13, and IL-17 ELISA kits were obtained from eBioscience. IL-10 ELISA kit was purchased from BD Biosciences.

### Cell isolation

Spleen and lymph nodes (axillary, inguinal) were removed from mice and gently meshed in DMEM containing 10% FBS to prepare the single cell suspensions. CD4^+^CD25^+^ Tregs and CD4^+^CD25^−^ T cells were separated by the CD4^+^CD25^+^ regulatory T cell isolation kit from Miltenyi Biotec, per manufacturer's protocols. Routinely, the CD4^+^CD25^−^ T and CD4^+^CD25^+^ Tregs subpopulations were >90% and >80–85%, respectively.

### CFSE labeling of T cells

1×10^6^ cells/ml purified cells were labeled at with 5 μM CFSE (Invitrogen) in pre-warmed PBS containing 0.1% BSA, incubated at 37°C for 10 mins, then washed in fresh medium for three times. Set up *in vitro* cell cultures under appropriate conditions as indicated in figures.

### Cell proliferation

Splenocytes were stimulated with 0.5 μg/ml soluble anti-CD3 antibody, anti-CD3 plus 2 μg/ml soluble anti-CD28 antibodies, or in the presence of 5 ng/ml IL-2 for three days. For purified cells, CD4^+^CD25^−^ T and CD4^+^CD25^+^ Tregs were stimulated by 0.5 μg/ml soluble anti-CD3 antibody and T-cell depleted splenocytes as antigen-presenting cells (APCs) for three days. In some experiments, the purified cells were stimulated by 5 μg/ml coated anti-CD3 and 2 μg/ml soluble anti-CD28 antibodies without APCs. The cells were labeled with 1 μCi/well tritiated thymidine (^3^H-TdR) for the final 16 hrs of culture, harvested and counted in a liquid scintillation and luminescence counter 1450 Microbeat TRILUX. In some experiments the cells were labeled with CFSE before culture, at indicated days after stimulation, the cells were collected for staining. The APCs were irradiated with Gammacell 1000 at the dose of 3000 rad and washed three times with DMEM plus 10% FBS and used for proliferation assays.

### Co-culture of CD4^+^CD25^+^ T and CD4^+^CD25^−^ T cells

Freshly isolated CD4^+^CD25^−^ T cells (5×10^4^/well) from spleens of PARP-1^−/−^ or age-matched WT mice were stimulated by 0.5 μg/ml soluble anti-CD3 antibody and APCs (2×10^5^/well) in the presence or absence of freshly isolated indicated number of CD4^+^CD25^+^ Tregs in U-bottom 96-well plates. The cells were cultured at 37°C and 5% CO_2_ for three days. The cells were labeled with 1 μCi/well ^3^H-TdR for the final 16 hrs, harvested and counted in a liquid scintillation and luminescence counter 1450 Microbeat TRILUX. In some experiments, 1 μM TGF-β RI kinase inhibitor II or 10 ng/ml anti-IL-10 mAb was added into the culture system. For some experiments, CD4^+^CD25^−^ T cells or CD4^+^CD25^+^ Tregs were labeled with CFSE before culture and, after the indicated days, collected to stain surface molecules and intracellular Foxp3, analyzed proliferation by flow cytometry.

### Cytokine induction and determination

The cells were cultured with antibodies which were indicated in different groups as described above. Cell supernatants were collected at indicated days, and then determined the IL-2, IL-4, IL-10, IFN-γ, IL-6, IL-13, IL-17, and TNF production by ELISA Kits using manufacturer's procedure.

### Flow cytometry analysis

Single cell suspensions were prepared from spleen and lymph nodes, or cultured isolated cells (1×10^6^) were re-suspended in flow cytometry buffer (PBS containing 0.5% BSA). Cells were stained with anti-CD4, CD8, and CD25 antibodies on ice for 30 mins in dark. Cells were washed twice with flow cytometry buffer, then collected and analyzed by flow cytometry. For Foxp3 staining, cells which were stained with surface markers were fixed and permeabilized with Fixation/Permealization kit (eBioscience) according to the manufacturer's suggested protocol and incubated with anti-Foxp3 antibody at 4°C for 30 mins in the dark. Cells were then washed and analyzed by flow cytometry (FACSCalibur, BD Biosciences), which was also used to assess cell divisions in CFSE-labeled cells.

### Real-time PCR

Mouse *Foxp3* and *Hprt* were analyzed with TaqMan gene expression assay kit. Primers for the following were from Applied Biosystems: mouse *Foxp3*, Mm00475156; *Hprt*, Mm00446968.

### Chromatin immunoprecipitation assay (ChIP)

ChIP assays were performed using a Red ChIP kit (Diagenode Inc. NJ, USA) according to the manufacturer's instructions. Briefly, CD4^+^CD25^+^ Tregs (5×10^6^) were isolated from the spleen and lymph nodes of PARP-1^−/−^ and WT control mice. Cells were then fixed with 1% formaldehyde and chromatin was fragmented using a bioruptor^TM^ UDC-200 (Diagenode Inc.). Equal amounts of processed chromatin per sample were used as input controls or incubated with 4 µg of the corresponding antibodies: anti-Foxp3 (F-9, Santa Cruz) or respective control antibody (Mouse IgG1 antibody (107.3), BD Biosciences) bound to A/G pre-bound agarose beads. DNA-immunoprecipitates were purified and extracted using a PCR-DNA purification kit (Qiagen). Immunoprecipitated and total input DNAs were analyzed using a SYBR-Green Supermix kit (BIO-RAD) and a Quantitative real-time PCR icycler iQ^TM^ detection system (BIO-RAD). The PCR primers used to detect mouse Foxp3 conserved non cording DNA sequence element 2 (CNS2) were: Foxp3-CNS2-amplicon, (Forward) 5′-GGCTTTAGGTGGTTCCCATT-3′, (Reverse) 5′-AAGGTTGGATGCTTGGTGAG-3′.

### Statistical analysis

Statistical significance of differences was determined by Student's *t* test (two-tailed test).

## Results

### Deletion of PARP-1 enhances immunosuppressive function of Foxp3^+^ Tregs

**Figure 1:**
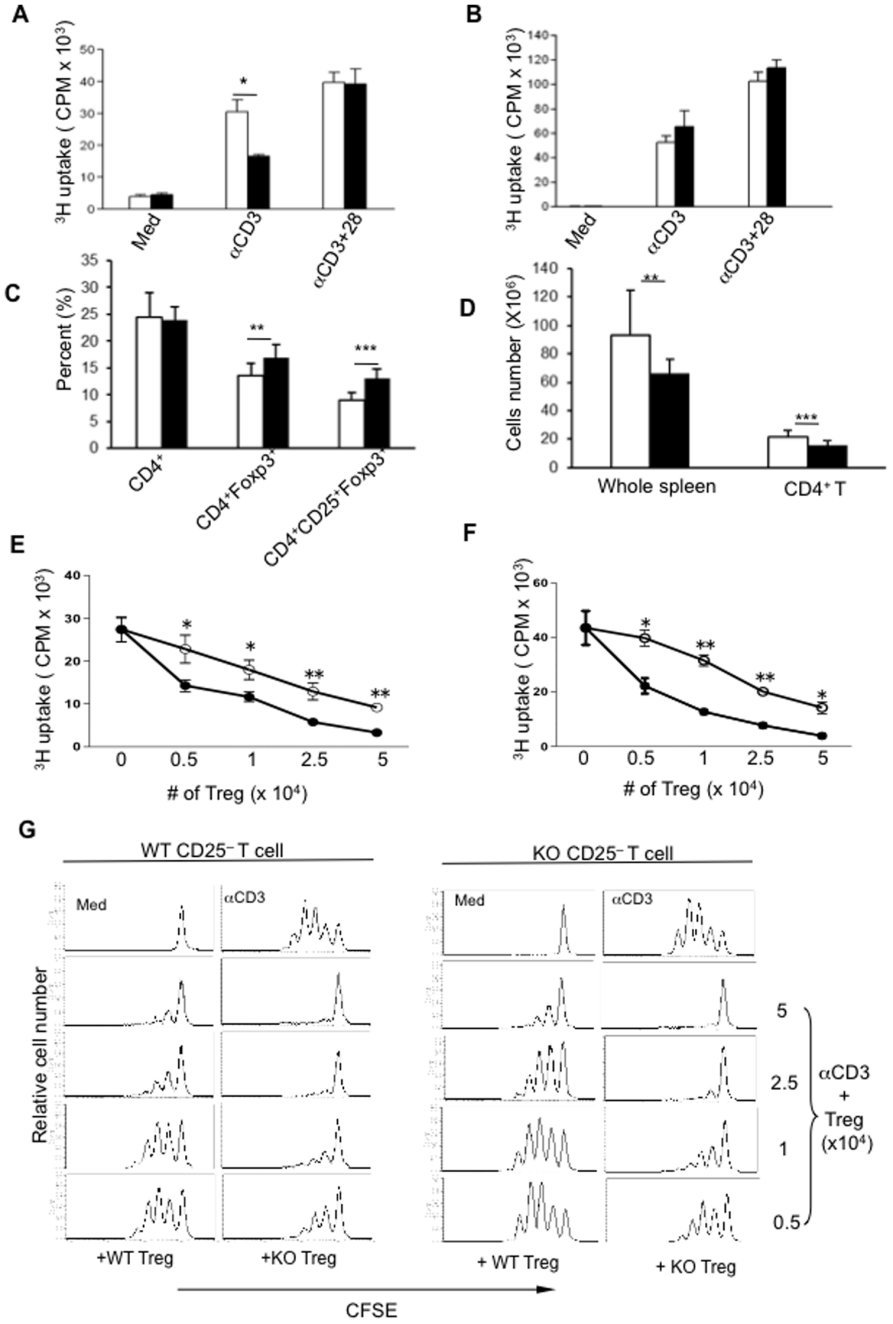
Deletion of PARP-1 confers stronger immunosuppressive function in Tregs. Whole spleen cells (**A**) or purified CD4^+^CD25^−^ T cells (**B**) from PARP-1^−/−^ and WT control mice were stimulated with soluble anti-CD3 antibody (**A**) or plate-coated anti-CD3 antibody (**B**) or plus soluble anti-CD28 antibody. Cell proliferation was measured by ^3^H-TdR uptake after 3 days culture. (**C**) The frequencies of CD4^+^, CD4^+^Foxp3^+^ and CD4^+^CD25^+^Foxp3^+^ T cells in spleen of PARP-1^−/−^ and WT control mice (WT, white bar, n = 15 mice; PARP-1^−/−^, black bar, n = 13 mice) were analyzed by flow cytometry. (**D**) The cells number of whole spleen cells and splenic CD4^+^ T cells PARP-1^−/−^ and WT control mice (WT, white bar, n = 15 mice; PARP-1^−/−^, black bar, n = 13 mice). (E^−^F) CD4^+^CD25^+^ Tregs from PARP-1^−/−^ and control WT mice (WT, white circle; PARP-1^−/−^, black circle) were cultured with freshly isolated WT CD4^+^CD25^−^ (**E**, 5×10^4^) or PARP-1^−/−^ CD4^+^CD25^−^ (**F**, 5×10^4^) T cells in the presence of WT splenic APCs at indicated cell numbers. T cells proliferation was determined by ^3^H-TdR uptake after 3 days. (**G**) CFSE-pre-labeled WT (left two column graphs) or PARP-1^−/−^ (right two column graphs) CD4^+^CD25^−^ T cells (5×10^4^) were cultured with indicated numbers of CD4^+^CD25^+^ Tregs in the presence of WT splenic APCs for 3 days. The cells were then stained with anti-CD4 antibody, and CD4^+^CFSE^+^ cells were gated and displayed as histograms by flow cytometry. The data are shown as Mean±SD Experiments were performed 3–5 times, *p<0.05, **p<0.01, ***p<0.005.

**Figure 2:**
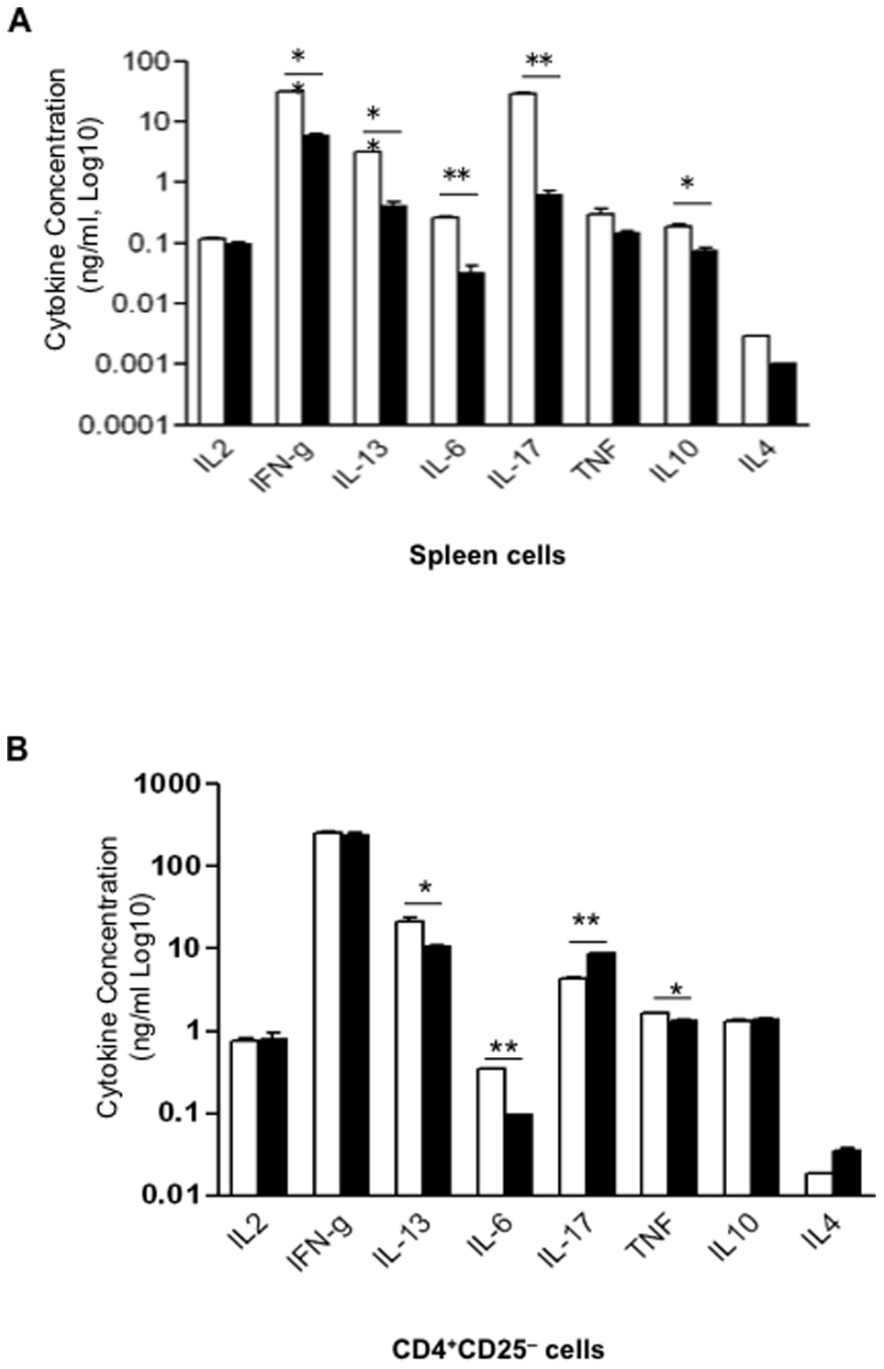
Cytokines production is lower in PARP-1^−/−^ T cells. (**A**) The profile of indicated cytokines in the supernatants of PARP-1^−/−^ (black bar) and control WT (white bar) whole spleen cells stimulated with soluble anti-CD3-antibody (0.5 µg/ml) for one (IL-2, IL-4), two (TNF) and three (others) days. (**B**) Cytokine production in purified CD4^+^CD25^−^ T cells stimulated with plate-coated anti-CD3 (5 µg/ml) for the same time as A. The cytokines were determined with ELISA assay. The data are shown as Mean±SD of duplicate measurements and representative of 3–4 experiments, **p<0.01, *p<0.05.

Given the fact that gene deletion or protein activity inhibition of PARP-1 suppresses immune responses and inflammation, we reasoned that PARP-1 deficiency might affect T cell responses to TCR stimulation. We stimulated PARP-1^−/−^ T cells with anti-CD3 antibody, and observed that PARP-1^−/−^ T cells showed significantly lower levels of proliferation (**Fig. 1A**) and cytokine production (**Fig. 2A**) upon anti-CD3 antibody stimulation. The decrease in T cell proliferation was reversed when anti-CD28 antibody (**Fig. 1A**) and/or exogenous IL-2 (data not shown) were added into the cultures, suggesting that PARP-1^−/−^ T cells were either energized and/or super-suppressed by Tregs. To distinguish these possibilities, we first depleted CD25^+^ Tregs from CD4^+^ T cells and observed that PARP-1^–/–^ CD4^+^CD25^–^ T cells showed a similar, or even higher, level of proliferative response to TCR stimulation compared to WT control CD4^+^CD25^–^ T cells (**Fig. 1B**). The majority of cytokines were also restored ((**Fig. 2B**). We analyzed the percentage and cell numbers in splenocytes of PARP-1^–/–^ and WT mice, and found that the frequency of CD4^+^Foxp3^+^ Tregs, especially CD4^+^CD25^+^Foxp3^+^ Tregs, significantly increased in PARP-1^−/−^ mice (**Fig. 1C**). Since PARP-1^−/−^ mice had decreased total splenocytes **(** **Fig. 1D** **)** and CD4^+^ T cells **(** **Fig. 1D** **),** there was no difference of absolute number of splenic CD4^+^CD25^+^Foxp3^+^ Tregs between PARP-1^–/–^ and WT control mice (data not shown). We then examined Tregs function in the standard co-culture suppression system *in vitro* [28], [29]. Strikingly, PARP-1^–/–^ Tregs showed significantly stronger suppression of TCR-driven responder T cell proliferation than did WT Tregs (**Fig. 1E–F**). This enhanced suppression occurred at all ratios of Tregs to the effector cells and irrespective of the source of the responder T cells population. PARP-1^–/–^ Tregs showed more suppressive activity to both WT (**Fig. 1E**) and PARP-1^–/–^ (**Fig. 1F**) CD4^+^CD25^–^ responder T cells compared to WT Tregs. This up-regulation of suppression was also independent of the source of antigen presenting cells (APCs) (data not shown) suggesting an intrinsic functional change in Tregs in the deficiency of PARP-1. To further confirm enhanced suppression of PARP-1^–/–^ Tregs to responder T cells, we used CFSE-labeled responder CD4^+^CD25^−^ T cells. Consistent with the ^3^H-TdR thymidine incorporation assay, PARP-1^–/–^ Tregs indeed showed a much stronger suppression of responder T cell proliferation (**Fig. 1G**). Thus, deletion of PARP-1 confers a greater suppressive function of Foxp3^+^ Tregs.

### Deletion of PARP-1 enhances and stabilizes Foxp3 expression in Tregs

We next sought to understand the molecular mechanisms responsible for the enhanced suppressive function of PARP-1^–/–^ Tregs. PARP-1^−/−^ Foxp3^+^ Tregs showed similar levels of Treg-associated molecules such as CTLA-4 and GITR (data not shown). We then examined whether TGF-β1 and IL-10 were involved in Tregs-mediated suppression by including of TGF-β receptor I (TβRI) kinase inhibitor or anti-IL-10R antibody into the co-cultures. Blockade of TGF-β signaling partially reversed PARP-1^–/–^ Tregs suppression as did for WT Tregs, whereas blockade of IL-10 signaling had no effect (data not shown), suggesting that TGF-β signaling plays only a part role in PARP-1^−/−^ Tregs suppression.

**Figure 3:**
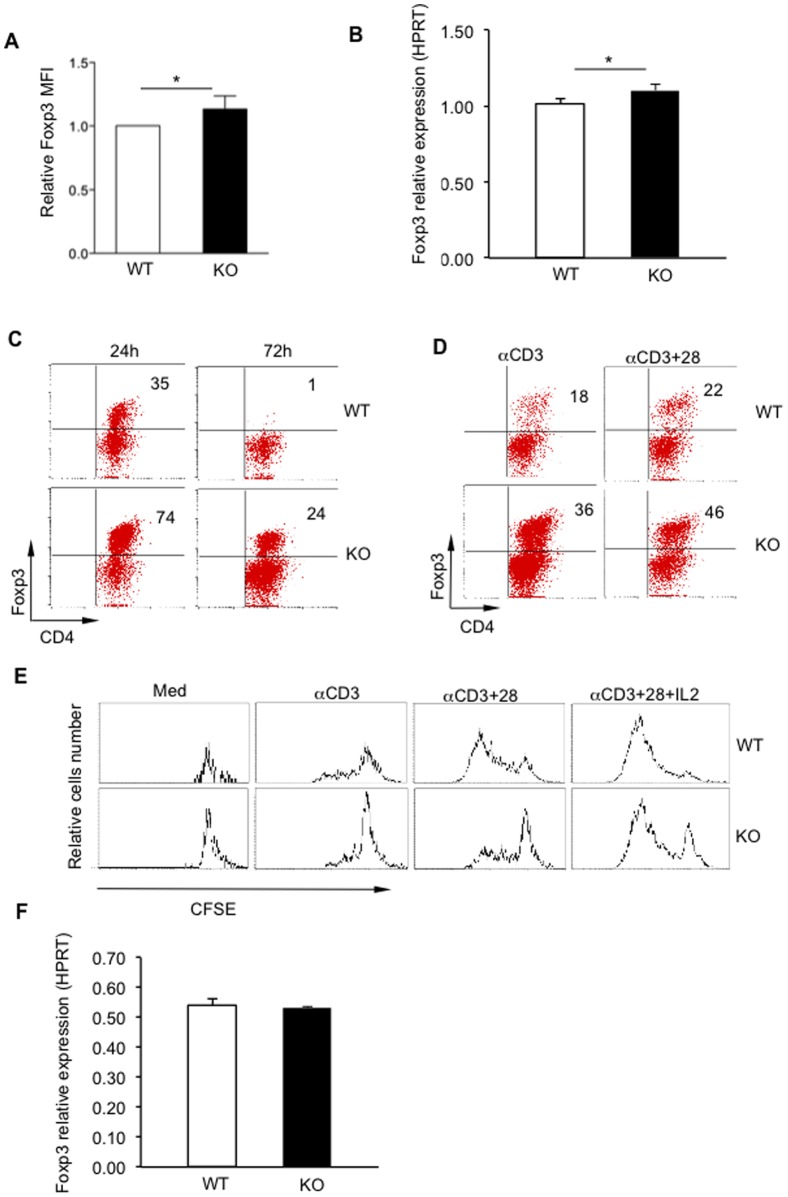
PARP-1^−**/**−^ Tregs show higher and more stable Foxp3 expression. (**A**) Fresh isolated CD4^+^CD25^+^ Tregs were stained with CD4 and Foxp3 antibodies (WT Tregs: 83–87% Foxp3^+^; PARP-1^−/−^ (KO) 85–90% Foxp3^+^). CD4^+^Foxp3^+^ cells were gated with flow cytometry and the mean fluorescence intensity (MFI) of Foxp3 expression is determined. To normalize for experimental variation between experiments, data were normalized relative to WT Tregs MFI used as 1 in each experiment. P<0.05, n = 7. (**B**) *Foxp3* mRNA expression was analyzed in fresh isolated CD4^+^CD25^+^ Tregs from PARP-1^−/−^ or WT mice. Data shown are representative of four independent experiments. *p<0.05. (**C)** CD4^+^CD25^+^ Tregs from PARP-1^−/−^ (KO) or WT mice were cultured in serum-free medium (X-vivo 20) for 24 hrs or 72 hrs in the absence of exogenous stimuli and cytokines. After culture, cells were collected and then stained with anti-CD4 and Foxp3 antibodies and analyzed by flow cytometry. (**D**) CD4^+^CD25^+^ Tregs were cultured with plate-coated anti-CD3 antibody alone or plus soluble anti-CD28 antibody for 3 days and then stained with anti-CD4 and Foxp3 antibodies and analyzed by flow cytometry. Data shown are representative experiment of two times. (E) CFSE pre-labeled CD4^+^CD25^+^ Tregs were cultured with plate coated anti-CD3 antibody alone, anti-CD3 plus CD28 antibodies or in the presence of IL-2 for three days. CD4^+^Foxp3^+^T cells were gated to assess the CFSE dilution. Data shown are representative of two independent experiments. (**F**) *Foxp3* mRNA expression was analyzed in CD4^+^CD25^+^ Tregs from PARP-1^−/−^ or WT mice which were stimulated with anti-CD3 and CD28 antibodies for overnight. The mice were backcrossing with C57BL/6 mice for at least twelve generations.

We then examined the levels of Foxp3 expression in PARP-1^–/–^ Tregs, as it has been demonstrated that sufficient levels of Foxp3 expression in Tregs is a pre-requisite for ensuring their suppressive function [16], [17]. To this end, we showed that even freshly isolated PARP-1^–/–^ CD4^+^Foxp3^+^ Tregs expressed significantly higher amounts of Foxp3 protein on single cell, as determined by its mean fluorescence intensity (MFI) compared to WT counterparts (**Fig. 3A**), indicating that deletion of PARP-1 enhances Foxp3 protein in Tregs. The increased Foxp3 expression in PARP-1^–/–^ Tregs was also found at mRNA level (**Fig. 3B**).

We then determined whether the lack of PARP-1 influenced the stability of Foxp3 expression in Tregs. We cultured Tregs in a serum-free medium (X-VIVO-20) in the absence of any exogenous cytokines, growth factors and serum to exclude completely the potential effects of these factors. We showed that PARP-1^–/–^ Tregs exhibited stronger and more stable Foxp3 expression. For example, while only 30% of the live WT Tregs expressed Foxp3 at 24 hrs after culture, more than 70% of viable PARP-1^–/–^ Tregs still had Foxp3 expression (**Fig. 3C**). Even at 72 hrs after culture, there were still about 20–30% PARP-1^–/–^ Tregs expressing Foxp3, whereas there was almost no WT Tregs showing Foxp3 (<1%) (**Fig. 3C**). A similar trend was observed when Tregs were cultured in complete DMEM medium containing 10% FBS (data not shown). The data indicate that Tregs deficient in PARP-1 are inherently more resistant to Foxp3 loss in the steady state and in a growth factor-depriving environment.

PARP-1^–/–^ Tregs also showed more stable Foxp3 expression in response to TCR stimulation. When cultured with plate-bound anti-CD3 antibody alone or plus soluble anti-CD28 antibody, PARP-1^–/–^ Tregs showed more Foxp3 expression compared to WT Tregs (**Fig. 3D**). Intriguingly, PARP-1^−/−^ Tregs showed substantially less proliferative response in response to TCR stimulation compared to WT Tregs (**Fig. 3E**), which was inversely associated with their Foxp3 levels (**Fig. 3D**). Intriguingly, however, the there was no difference of *Foxp3* mRNA between PARP-1^−/−^ Tregs and WT Tregs after overnight culture with anti-CD3 and CD 28 antibodies (Fig.****3F). Addition of anti-CD28 antibody and exogenous IL-2 only partially correct the defect of proliferation in PARP-1^–/–^ Tregs (**Fig. 3E**). These data collectively indicate that PARP-1^–/–^ Tregs are resistant to TCR-induced Foxp3 loss, which may result in their decreased proliferative response upon TCR stimulation. Alternatively, but not exclusively, the decreased expansion of PARP-1^–/–^ Tregs was likely due to an inherent feature of these Tregs in the absence of PARP-1.

To further elucidate the Foxp3 expression and behavior of PARP-1^–/–^ Tregs when they interact with responder T cells in co-cultures, Tregs were pre-labeled with CFSE before co-culture with CD4^+^CD25^–^ responder T cells in the presence of APCs. Significantly, PARP-1^–/–^ Tregs showed substantially less proliferation compared to their WT Tregs (data not shown), in which the responder T cells were inhibited more intensively than those co-cultured with WT Tregs (**Fig. 1**). These data demonstrate that PARP-1^–/–^ Tregs show slower proliferative response even in the co-cultures with responder T cells, consistent with their low division in response to TCR stimulation in the absence of responder T cells (**Fig. 3E**). The data suggest that the more potent suppressive activity of PARP-1^–/–^ Tregs was unlikely due to their over-proliferation.

**Figure 4:**
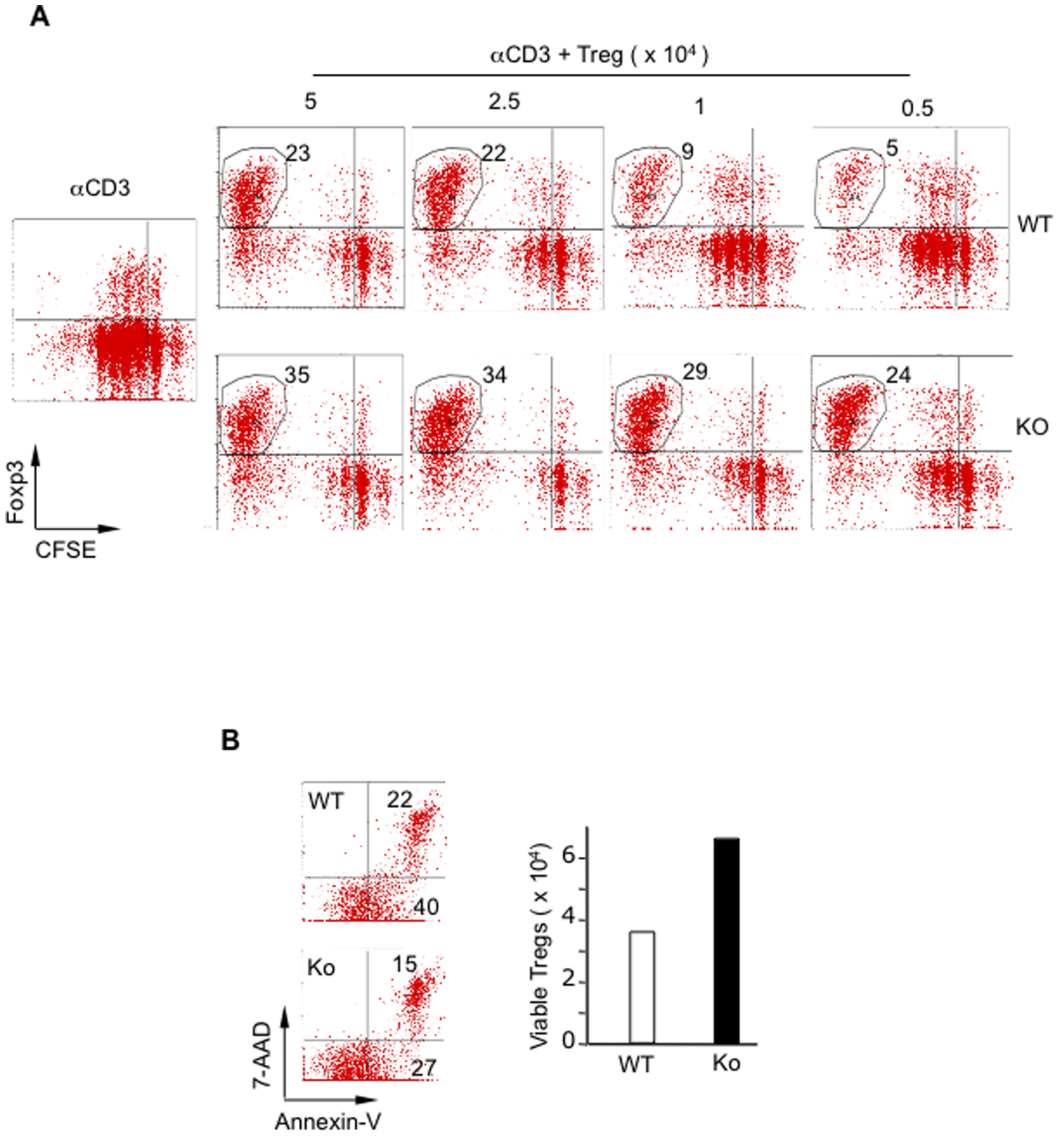
PARP-1^−/−^ Tregs showed increased survival in co-culture assay. **(A)** Enhancement of PARP-1^−/−^ Foxp3^+^ Tregs in the co-culture assay with CD4^+^CD25^−^ T cells. CFSE labeled WT CD4^+^CD25^−^ T cells (5×10^4^) were stimulated with anti-CD3 antibody and WT T-cell depleted splenic APCs in the absence and presence of indicated WT (top panel) or PARP-1^−/−^ (bottom panel) CD4^+^CD25^+^ Tregs at indicated cell numbers for 3 days. Cells were collected and then stained with anti-CD4 and Foxp3 antibodies and analyzed by flow cytometry. The dot plots are shown as the profile of Foxp3 vs. CFSE in gated live CD4^+^ T cells. The numbers indicate the percentage of CD4^+^Foxp3^+^ Tregs. The data are shown as a representative experiment of two times. (**B**) Resistance of PARP-1^−/−^ CD4^+^CD25^+^ Tregs toward activation-induced death. Purified CD4^+^CD25^+^ Tregs were cultured with plate-coated anti-CD3 and soluble anti-CD28 antibodies for 1 day and stained with Annexin-V and 7-AAD for apoptotic cells. Left panel, flow cytometry of WT (top) and PARP-1^−/−^ (KO, bottom) CD4^+^CD25^+^ Tregs. The numbers in the right quadrants indicate the early apopototic (Annexin-V^+^ 7-AAD^−^, bottom) and late apoptoic/dead (annexin-V^+^ 7-AAD^+^, top) cells. Right panel is the absolute number of viable Tregs (original input number 1×10^5^) after culture. The data shown are representative of three experiments.

Strikingly, despite their lower levels of proliferative response, the number of remaining PARP-1^–/–^ Foxp3^+^ Tregs was dramatically higher than that of corresponding WT Tregs in co-culture conditions (**Fig. 4A**). At a ratio of 1∶1 (Tregs to responder T cells) co-culture conditions, PARP-1^−/−^ Foxp3^+^ Tregs constituted 35% of live CD4^+^ cells, whereas WT Tregs only constituted 23% of live CD4^+^ cells (**Fig. 4A**). This difference of the remaining Tregs to responder T cell ratio in co-cultures increased between PARP-1^–/–^ and WT Tregs with reduction of initially added Tregs (**Fig. 4A**). Importantly, the majority of remaining Tregs expressed Foxp3. The accumulation of PARP-1^−/−^ Foxp3^+^ Tregs in these co-cultures could be due to their resistance to cell death, as they showed less apoptotic/necrotic cell death in the absence or presence of TCR stimulation as determined by Annexin-V and 7-AAD (**Fig. 4B**). In addition to Foxp3, PARP-1^−/−^ Tregs also expressed higher levels of CD25 in freshly isolated Tregs (data not shown) and also in response to TCR stimulation in the culture (data not shown). Thus, the deletion of PARP-1 stabilizes Foxp3 expression with less proliferative potential yet increased survival, although whether the stable Foxp3 expression confers Tregs resistance to death remains to be elucidated. Nonetheless, the accumulation of these knockout Tregs form not only a huge “sink” for consuming limited amounts of IL-2 at the expense of responder T cell proliferation, but also a potent population of cells actively suppressing the expansion of responder T cells.

### PARP-1 deficiency increased Foxp3 binding at the CNS2 region

**Figure 5:**
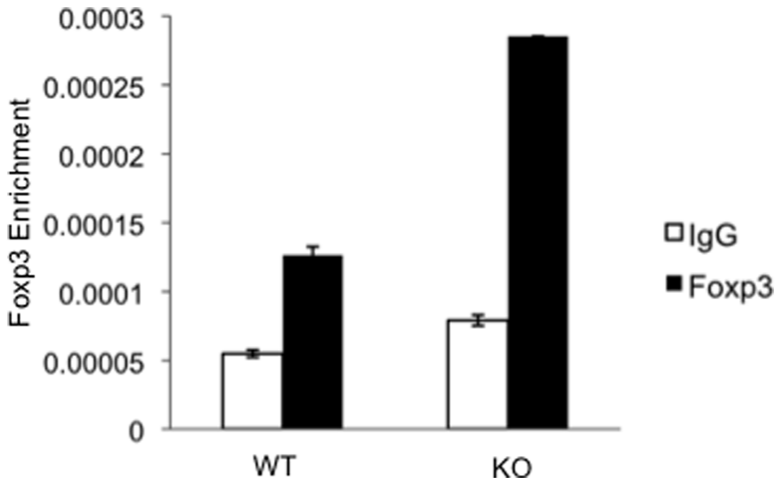
Foxp3 binds more at CNS2 region in PARP-1^−/−^ Tregs. Freshly isolated CD4^+^CD25^+^ Tregs from the spleen of PARP-1^−/−^ and WT control mice were fixed and chromatin was fragmented. Equal amounts of processed chromatin per sample were used as input controls or incubated with 4 µg of the corresponding antibodies: anti-Foxp3 (black bars) or respective control antibody (white bars) bound to A/G pre-bound agarose beads. Immunoprecipitated and total input DNAs were analyzed by using real-time PCR. The data are representative of two independent experiments.

We next explored the molecular mechanisms responsible for the high and stable Foxp3 expression in Tregs in the absence of PARP-1. Recent studies indicated that the conserved non-coding DNA sequence (CNS) element 2 (CNS2) at the *Foxp3* locus in Tregs is required for Foxp3 stabilization. It was suggested that Foxp3 recruitment to this region facilitates the heritable maintenance of the active state of the *Foxp3* locus and, therefore, Tregs lineage stability [18]. As PARP-1 deficiency increased the amount and stability of Foxp3, we hypothesized that the CNS region bound more Foxp3 in PARP-1^–/–^ Tregs. To test this hypothesis, we determined the Foxp3 binding at the CNS2 region in freshly isolated CD4^+^CD25^+^ Tregs by chromatin immunoprecipitation–coupled quantitative PCR (ChIP-qPCR) assay. We showed that, indeed, PARP-1^−/−^ Tregs had substantially higher levels of Foxp3 binding to the CNS2 on the *Foxp3* locus than did WT Tregs (**Fig. 5**), suggesting that PARP-1 normally prevents/inhibits Foxp3 binding at the CNS2 region, destabilizing Foxp3 expression in Tregs.

## Discussion

We have demonstrated that deletion of PARP-1 enhanced Foxp3^+^ Tregs-mediated immunosuppression. In contrast to a recent study showing PARP-1 deletion has no effects on Tregs function [30], we show increased suppressive activity of PARP-1^–/–^ Tregs by both ^3^H-TdR incorporation and CFSE-labeled assays. We have further elucidated that the enhanced suppression of PARP-1^−/−^ Foxp3^+^ Tregs is mainly attributed to their stable expression of Foxp3, suggesting a previously unrecognized role for PARP-1 in the regulation of Foxp3 expression in Tregs. The PARP-1 control of Foxp3 expression is likely attributed to its interference/suppression of Foxp3 binding to the CNS2 region at the *Foxp3* locus in Tregs, as deletion of PARP-1 substantially enhanced the CNS2 recruitment of *Foxp3* binding. How PARP-1 influences Foxp3 binding at the CNS2 remains to be elucidated. PARP-1 has been reported to be involved in epigenetic chromatin structure and function [31], and/or PARP-1 may bind to the *Foxp3* gene to negatively regulate its expression, although no evidence is available for the binding sequence of the PARP-1 DNA binding sequence. PARP-1 may also regulate *Foxp3* gene indirectly through interaction with other transcription factors. Nonetheless, the stable and persistent expression of Foxp3 in the absence of PARP-1 confers a stronger Tregs suppressive activity. It remains to be elucidated, however, how stable Foxp3 expression allows Tregs to confer higher immunosuppressive activity, as Foxp3 is a nuclear transcription factor and is not expressed on the surface of Tregs. Foxp3 was reported to promote CTLA-4 expression on Tregs [32], but CTLA-4 expression was similar between the PARP-1^−/−^ and WT Tregs, suggesting CTLA-4 is unlikely involved in the process. TGF-β1 plays a partial role in the suppression, as blockade of TGF-β signaling only partially reversed the suppression, and this effect is not limited to PARP1^−/−^. IL-10 is not involved, as anti-IL-10R neutralization antibody has no effect on PARP-1^–/–^ Tregs-mediated suppression, at least *in vitro*. It is likely that the higher levels of CD25 is involved in the suppression, as it could serve as a sink for the limited IL-2 produced by the responder T cells as demonstrated *in vitro* and may also be the case *in vivo*. Nonetheless, the stable and persistent Foxp3 expression in Tregs in the absence of PARP-1 is likely a main factor for conferring their stronger immunosuppressive activity.

Importantly, we showed that deletion of PARP-1 leads to increased Foxp3 binding to the CNS2 region at the *Foxp3* locus in Tregs. The increased Foxp3 binding might not only stabilize the Fxop3 levels in naive PARP-1^−/−^ Tregs, but also help maintain Foxp3 expression in TCR-stimulated PARP-1^−/−^ Tregs (Fig.****3D), which might not be necessarily involved *Foxp3* mRNA transcription (Fig.****3F). In addition, it is also likely that PARP-1 deficiency increases the stability of Foxp3 protein itself, which remains to be elucidated. Nevertheless, this finding is in line with the recent identification of CNS2 as a crucial region of the *Foxp3* gene for Foxp3 stabilization and expression, and provides evidence for the first transcription factor that may participate in regulation of Foxp3 binding onto the CNS2 locus. Our data provides a starting point for further elucidation of a network of factors regulating Foxp3 binding onto the CNS2 region and participating in *Foxp3* gene stabilization. Our findings may help further understand the mechanisms of Foxp3^+^ Tregs mediated immune regulation.
